# Research priorities for accelerating the achievement of three 95 HIV goals in Cameroon: a consensus statement from the Cameroon HIV Research Forum (CAM-HERO)

**DOI:** 10.11604/pamj.2021.40.124.31068

**Published:** 2021-10-29

**Authors:** Anastase Dzudie, Boris Tchounga, Rogers Ajeh, Charles Kouanfack, Peter Vanes Ebasone, Tatiana Djikeussi, Léonard Bonono Nyoto, Joseph Fokam, Jérôme Ateudjieu, Patrice Tchendjou, Ezechiel Ngoufack Jagni Semengue, Fabrice Youbi Kamgang, Jean Anoubessi, Marie Varloteaux, Boris Youngui, Felicite Naah Tabala, Benjamin Atanga, Leonie Simo, Armel Zemsi, Emile Nforbih Shu, Gilles Ndayisaba, Annereke Nyenti, Apungwa Cornelius Ntabe, Therese Abong Bwemba, Eugene Sobngwi, Serge Clotaire Billong, John Ditekemena, Anne Cecile Zoung-Kanyi Bissek, Louis Richard Njock

**Affiliations:** 1 Clinical Research Education, Networking and Consultancy, Yaoundé, Cameroon,; 2 Faculty of Medicine and Biomedical Sciences, University of Yaoundé I, Yaoundé, Cameroon,; 3 Service of Internal Medicine and Subspecialities, Douala General Hospital, Douala, Cameroon,; 4 Lown Scholars Program, Department of Global Health and Population, Harvard T. H. Chan School of Public Health, Boston, USA,; 5 Elizabeth Glaser Pediatric AIDS Foundation, Yaoundé, Cameroon,; 6 HIV Day Hospital, Yaoundé Central Hospital, Yaoundé, Cameroon,; 7 Faculty of Medicine and Pharmaceutical Sciences, University of Dschang, Dschang, Cameroon,; 8 Department of Medicine, University of Cape Town, Cape Town, South Africa,; 9 National AIDS Control Committee, Ministry of Public Health, Yaoundé, Cameroon,; 10 International Reference Centre Chantal Biya (IRCCB), Yaoundé, Cameroon,; 11 Faculty of Health Sciences, University of Buea, Buea, Cameroon,; 12 Division of the Fight against Diseases, Ministry of Public Health, Yaoundé, Cameroon,; 13 Cameroon office, National Agency for Research on AIDS (ANRS), Yaoundé, Cameroon,; 14 Division of Health Operational Research, Ministry of Public Health, Yaoundé, Cameroon,; 15 Limbe Regional Hospital, Limbe, Cameroon,; 16 Cameroon Bioethics Initiative, Yaoundé, Cameroon,; 17 National Ethics Committee, Yaoundé, Cameroon,; 18 General Secretariat, Ministry of Public Health, Yaoundé, Cameroon

**Keywords:** Treat all, HIV/AIDS, Cameroon, research priorities

## Abstract

**Introduction:**

the Treat-All remains the globally endorsed approach to attain the 95-95-95 targets and end the AIDS pandemic by 2030, but requires some country-level contextualization. In Cameroon, the specific research agenda to inform strategies for improving HIV policy was yet to be defined.

**Methods:**

under the patronage of the Cameroon Ministry of health, researchers, policy makers, implementing partners, and clinicians from 13 institutions, used the Delphi method to arrive at a consensus of HIV research priorities. The process had five steps: 1) independent literature scan by 5 working groups; 2) review of the initial priority list; 3) appraisal of priorities list in a larger group; 4) refinement and consolidation by a consensus group; 5) rating of top research priorities.

**Results:**

five research priorities and corresponding research approaches, resulted from the process. These include: 1) effectiveness, safety and active toxicity monitoring of new and old antiretrovirals; 2) outcomes of Antiretroviral Therapy (ART) with focus in children and adolescents; 3) impact of HIV and ART on aging and major chronic diseases; 4) ART dispensation models and impact on adherence and retention; 5) evaluations of HIV treatment and prevention programs.

**Conclusion:**

the research priorities resulted from a consensus amongst a multidisciplinary team and were based on current data about the pandemic and science to prevent, treat, and ultimately cure HIV. These priorities highlighted critical areas of investigation with potential relevance for the country, funders, and regulatory bodies.

## Introduction

Ending the HIV/AIDS remains a global health priority. In 2015, the World Health Organization (WHO) recommended the HIV Treat All strategy as a global approach for the control of the HIV/AIDS pandemic [1]. This WHO policy recommended HIV Treat all approach and the United Nations Program on HIV/AIDS (UNAIDS) set goals to fast track the agenda; 95-95-95 targets to be attained by the year 2030 [2]. The three 95 targets stipulate that 95% of all people living with HIV will know their HIV status; 95% of people with diagnosed HIV infection will receive antiretroviral therapy (ART); and 95% of all people receiving ART will have suppressed viral load. Cameroon implemented the HIV treat all strategy nationwide in 2016 [3]. According to the 2018 Cameroon population based HIV impact assessment report [4]; only 46.9% of people living with HIV/AIDS (PLWHA) knew their status, 91.3% of those who knew their status were on ART and 80.0% of those on ART had viral suppression. The population level HIV prevalence was 3.7% and the population level viral suppression was 44.7%. These indicators highlighted a significant gap towards achieving the 95-95-95 target in 2020. This implies Cameroon needs extra efforts to close the gaps of 2020 and accelerate progress towards attaining the 95-95-95 targets by 2030. To attain these targets, the Cameroon Ministry of Health (MoH) needs to increase investment in areas of immediate and critical importance that can only be identified by contextual research and scientific evidences. This targeted translational research should address major barriers to HIV service uptake, including strategies to improve HIV testing uptake, linkage to care and treatment, retention in care and sustained viral suppression. Over the past 10 years, there is an increasing volume of HIV research in Cameroon, providing some useful scientific evidences in various areas of HIV care. However, like other health research areas, Cameroon capacity to support HIV research or to respond to funders is not unlimited and collaboration between researchers and between researchers and decision-makers of the MoH would facilitate more systematic investigations and much better response to the lack of evidence. Minding the need to improve national HIV collaborative research and to provide needful research evidence to inform national HIV program policies, a group of four leading HIV research organisations, working in closed collaboration and under the leadership of the Cameroon MoH, through its Division of Health Operational Research (DROS) and the National AIDS control committee (NACC), formed the Cameroon HIV research forum (CAM-HERO) in November 2020. One of the initial goals of this group was to establish HIV/AIDS research priorities for the country. This report presents the adopted national HIV research priorities list and the procedures used.

## Methods

We adopted the Delphi approach, a common used method to reach research consensus by experts in diverse fields, including health research [5-7]. The Delphi´s method generally constitutes a series of steps which allow elucidation and aggregation of opinion from a working group of researchers or experts [7]. The Delphi method allows experts to provide inputs continuously and independently, during each stage in the process, rendering the approach less liable to bias by preventing strong opinions from influencing weaker ones [5,6].

**Figure 1:**
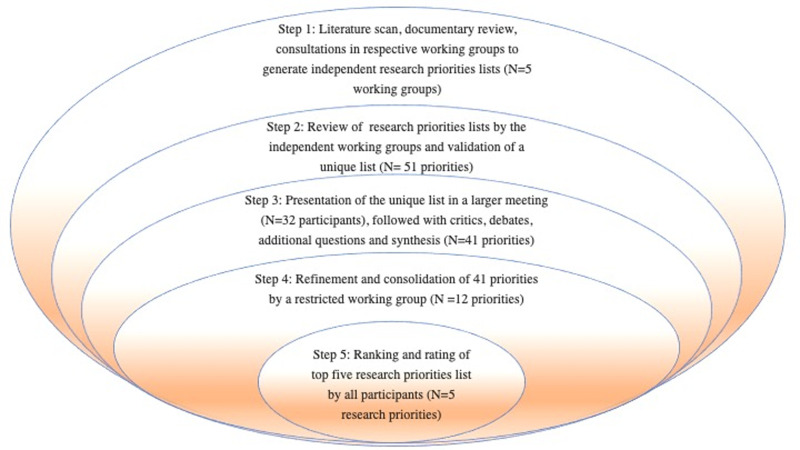
steps in the determination of research priorities

The CAM-HERO Kribi conference researchers went through five major steps to reach a consensual list of five priority HIV research areas, as illustrated in Figure 1. Lead investigators from the Elizabeth Glaser Pediatric AIDS Foundation (EGPAF) and the International epidemiology Databases to Evaluate AIDS/Clinical Research Education Networking and Consultancy research group (CRENC-IeDEA). CRENC-IeDEA (N=4) and EGPAF (N= 6) led the initial steps, supported by the Division of Health Operations Research (DROS) at the Cameroon MoH (N=3). As the process advanced, other actors working on diverse aspects of HIV research in Cameroon joined, including the National AIDS Control Committee (NACC) (N = 2), which is the arm of MoH in charge of HIV program coordination in Cameroon. Other major actors participated in the process, including the Cameroon office of ANRS, the French National Agency for AIDS Research (N=2), the Yaoundé Central Hospital HIV research group (N=1), and the Chantal Biya International HIV Research Centre (CIRCB, N=2).

### Step one: generation of initial research priority list by individual working groups

Leading Cameroon based HIV research implementing partners and other stakeholders were invited to participate in the process. The CRENC-IeDEA and EGPAF working groups did an initial thorough literature scan, documentary review and consultations with other HIV experts and generated their initial research priority lists of questions. Under each of the 95 targets, and according to the areas of expertise, the working group members used relevant key words to search and generate relevant literature published after 2010. The researchers also reviewed online annual reports of major HIV partners including the annual reports of the NACC, the Cameroon country reports of the WHO, UNAIDS, CDC amongst others. Other leading HIV research and implementing partners joined the process and independently generated their HIV research priority lists during the same period. This led to a total of five working groups (N=5), representing the five research partners or institutions.

### Step two: individual working group validation of research priorities

The generated lists from each of the working group were reviewed and validated by a bigger group in their respective organizations through interactive debates and discussions. This step ended with a validated list of priority research questions from each of the five working groups, summing up to a total of fifty-one research questions (N=51).

### Step three: review and validation of research priorities in a larger meeting

During a general meeting, each of the five [8] working groups presented their respective research priorities to an audience of 32 researchers and stakeholders from 13 institutions including; policy makers from the Ministry of Health, researchers from research institutions and universities, ethicists, national implementing partners and HIV clinicians from different hospitals. General comments and open interactive debates from the meeting participants followed each presentation. The working groups noted relevant comments, appraisals, and new questions that arose from the meeting participants and incorporated in their list. The coordination team also noted areas of overlapped between research priorities from the different working groups. At the end of this step, we adopted a step 3 list of research priorities (N=41).

### Step four: refinement and consolidation of research priorities

Through a series of sub meetings and teleconferences within and between working groups, we refined and consolidated the research priority list by identifying and synthesizing overlapping questions from different working groups. This led to a list of twelve research priorities (N=12).

### Step 5: rating of research priorities

This final step was done to validate consensus on the final list of research priorities and rank the priorities in terms of their pertinence to the HIV Treat All strategy in Cameroon. During this step, participants of the larger meeting (step three) completed an online survey on ranking and rating of the research priorities in two steps. At first participants ranked the top five priorities out of the 12 and secondly, they rated each priority on a five-point scale.

We used these two rating approaches to independently determine the top five research priorities. In the first approach we encouraged the ranking scoring while the second approach permitted us to choose the question with the highest rating whenever we had more than one research priorities with the same ranking score. Overall, we had a 4/5 (80%) concordance for the first top 4 priority questions. The 5^th^ question was selected through a consensus in a more restricted group (N=4 participants). Finally, for each research priority, examples of specific research questions were defined.

## Results

### Number and main backgrounds of participants

A total of 32 people participated in at least one round of the process and 26 people participated in at least 2 rounds. The participants had diverse HIV research backgrounds and were from 11 different institutions located in different regions in Cameroon. The final ranking surveys was responded to by 26 participants, including policy makers (N=5), HIV program implementing partners (N=6), senior level researchers (N=5), junior level researchers (N=3), HIV clinician doctors and research nurses (N=5), ethicist (N=2).

### Rating and consensus

The overall process to generate the list of top 5 research priorities by the group is presented in Figure 1. On further analysis to check for internal biases, we noted a general agreement (80%) between the top 5 priorities after the participants were stratified into two groups by level of experience and seniority in the field of HIV (expert group vs non expert group). The expert group had 100% agreement with the top 5 research priorities consensual list. The final research priority list which cut across different aspects of the HIV Treat All strategy are presented in Table 1. In total, 38 examples of specific research questions were defined for the 5 top priority research questions. Table 2, Table 2 (suite), Table 2 (suite 1), Table 2 (suite 2) illustrate the specific research question per research priority and suggest possible methods to achieve them.

**Table 1:** Cameroon HIV research priorities

No	Research priority	Ranking score (%)
	Monitor and assess ART effectiveness, safety and toxicity of old and new drugs: viral suppression rate, immunologic changes, toxicity and safety of newer ARVs including DTG based regimen in HIV population and pregnant women specifically, ARV resistances and early indicators, novel approaches to monitoring viral suppression	20/26(76.8)
	Assess short- and long-term outcomes of ART in children and adolescents including physical and psychosocial development, morbidity and mortality, care outcomes from infancy to adolescence	18/26(69.2)
	Assess the interactions and impact of HIV and ART on aging, non communicable diseases (NCDs) such as hypertension, cardiometabolic diseases and cancer, and on age-related morbidities like osteoarthritis or osteoporosis	16/26(61.5)
	Effectiveness of ART dispensation models and impact on short- and long-term ART adherence and retention: ART dispensation models including home based, Community based organization, private pharmacy, and multiple-month dispensation.	14/26(53.8)
	National surveys and evaluations of HIV treatment and prevention programs including: HIV test and treat strategy, self-testing, PrEP, cost effectiveness of interventions, sentinel surveillance of HIV disease burden, national AIDS spending, integrated surveys on priority populations)	12/26(46.2

**Table 2:** specific research questions per research priority

Example of research questions by priority	Methods
**Research priority 1**: Monitor and assess ART effectiveness, safety and toxicity of old and new drugs: viral suppression rate, immunologic changes, toxicity and safety of newer ARVs including DTG based regimen in HIV population and pregnant women specifically, ARV resistances and early indicators, novel approaches to monitoring viral suppression	
What are the viral suppression rate and determinants at different time points (6, 12, 24, and 36 months) in patients initiated on DTG based first and second line regimens disaggregated by age, sex and ART regimen, including key and priority populations groups? What are the most efficient strategies to improve viral suppression rate? What are the clinical outcomes of children and adolescents receiving ART, disaggregated by pediatric formulation (e.g.Lpv/r pellets, granules, raltegravir, etc.)? What are the new models/approaches to monitor viral suppression and how efficient are they? What are the proportions of patients on ART with treatment related site effects, and toxicity in new ARVs (e.g. DTG) disaggregated by age, sex, and medical status? What are the pregnancy outcomes (stillbirth, prematurity, birth weight, birth defects) in women receiving dolutegravir-based ART at the time of conception and during pregnancy? What are the proportions and determinants (sociodemographic and clinical) of treatment failure on first- and second line treatment and what proportions are changing lines and what is the time interval between failure and new line initiation? What are the clinical and viral outcomes of patients initiated on second or third line at 6 months, 12 months, 18 months, 24 months etc.? What is the percentage of patients eligible for resistance testing and what percentage had the test? What is the resistance pattern among patients on ART? What is the mortality rate and factors associated with early mortality (3-6 months of ART initiation) in patients on ART disaggregated by sex, age and treatment regimen? What are the quality indicators, including early signals, relevant to antiretroviral resistance Cameroon? What are the dynamics of resistance mutations within the era of transitioning to dolutrogravir based regimens? What are the differences in virologic response and acquired resistance profile in the use of DTG based regimens between first and 3rd line in Cameroon?	Prospective cohorts/Monitoring surveys/modelling Clinical trial Open cohorts Monitoring surveys Open cohorts Monitoring surveys Open cohorts Monitoring surveys Open cohorts Surveys Open cohorts

**Table 2(suite):** specific research questions per research priority

Exemple of research questions	Methods
**Research Priority 2**: Assess short- and long-term outcomes of ART in children and adolescents including physical and psychosocial development, morbidity and mortality, care outcomes from infancy to adolescence	
What is the evolution of compliance and secondary HIV treatment resistance in children and adolescents? What are the short- and long-term effects of HIV and ART exposure in children physical, psychological, cognitive development, morbidity and mortality? What is the progression of care HIV care cascade from infancy, adolescence to young adults? What is the incidence of HIV infection during adolescence? What is the survival of children who received PMTCT programs from birth to adolescence?	Clinical trials; Routine monitoring data; Open cohorts. Surveys. Open cohorts
**Research Priority 3:** Assess the interactions and impact of HIV and ART on aging, noncommunicable diseases (NCDs) such as hypertension, cardiometabolic diseases and cancer, and on age-related morbidities like osteoarthritis or osteoporosis	
What is the true burden of NCDs (hypertension, obesity, cancer, kidney diseases, diabetes mellitus) and age-related co-morbidities (osteoporosis, osteoarthritis) and association with HIV treatment outcomes in people aging with HIV. What are the specific ART and care needs of people aging with HIV? What is the prevalence and potential impact of interactions between ARVs and medications against prevalent NCDs (e.g., diabetic and anti-hypertensives)? What is the impact of HIV and ART on biological aging (fine lines and wrinkles, dullness of skin, dry skin, blotchiness, rough skin texture, and visible pores) as well as on age related quality of life (dependency, walking speed, emotional vitality, and subjective health)?	Routine monitoring data; Open cohorts

**Table 2(suite 1):** specific research questions per research priority

Research questions	Methods
**Research priority 4**: Effectiveness of ART dispensation models and impact on short- and long-term ART adherence and retention: ART dispensation models including home based, Community based organization, private pharmacy, and multiple-month dispensation	
**ART dispensation** How effective are old vs new community ART dispensation models (home base, CBOs, HIV support groups, private pharmacies, private clinics, religious organizations)? Including in a context of COVID-19? What is the impact of Differentiated models of care (DMoC) on short and long-term outcomes (retention, viral load suppression, resistance and death)? **ART adherence and retention** What is the ART adherence and retention rates, as well as their determinants in key populations (MSM, IVD, FSW) and vulnerable groups (children, prisoners, conflict zones)? What proportion of individuals retained in care at 6, 12, 24, 36+ months of ART, disaggregated by treatment regimen, sex and age group? What is the impact of public health interventions (peer mentorship, care decentralized and differentiated care models) on ART retention? How are facility- or program-level characteristics associated with adherence to national guidelines (for example, viral load monitoring or exploration of type of clinician providing services)?	Clinical trials Open cohorts
National surveys and evaluations of HIV treatment and prevention programs including: HIV test and treat strategy, self-testing, PrEP, cost effectiveness of interventions, sentinel surveillance of HIV disease burden, national AIDS spendings, integrated surveys on priority populations)	

**Table 2(suite 2):** specific research questions per research priority

Research questions	Methods
**Research priority 5:** National surveys and evaluations of HIV treatment and prevention programs including: HIV test and treat strategy, self-testing, PrEP, cost effectiveness of interventions, sentinel surveillance of HIV disease burden, national AIDS spendings, integrated surveys on priority populations)	
Which testing strategies are more efficient (e.g. self-testing, home-, and community-testing, etc.) in children and adolescents? What is the effectiveness of the HIV test and treat strategy? What is the effectiveness of HIV self-testing? What is the effectiveness of PrEP? What is the cost effectiveness of the major interventions to fight HIV? = To what extent are ARVs rationally used in health facilities? What is the evolution of HIV disease burden? What is the National AIDS Spending? How can behavioral and biological surveys be integrated in HIV priority populations?	Randomized control trial (RCT

### Research priority 1

Monitor and assess ART effectiveness, safety and toxicity of old and new drugs: viral suppression rate, immunologic changes, toxicity and safety of newer ARVs including DTG based regimen in HIV population and pregnant women specifically, ARV drug viral resistances and early indicators, novel approaches to monitoring viral suppression.

Context: short- and long-term success of ART programmes depend on the effectiveness and safety of antiretrovirals, especially newer molecules. Old and newer ARVs have been shown to have varying effectiveness in achieving viral suppression in urban and rural settings in in Cameroon. In 2018, a nationwide survey reported a 12-24 months viral suppression rate of 75.0% (65.2-82.7) and 67.7% (58.3-75.8) in urban and rural Cameroon settings [9]. An alarming rates of drug resistance (> 17%) have been reported in both adults and children within the first 24 months of ART in Cameroon [9,10]. These reports indicate an urgent need for a very close viral load monitoring while enhancing strategies to improve ART adherence and timely ART initiation. Dolutegravir (DTG), which is the WHO strongly recommended drug of choice for first line treatment of HIV since 2019 [11], was rollout in Cameroon at the beginning of 2020 [12]. DTG was recommended following compelling evidences from many studies on its effectiveness in achieving viral suppression, relative safety and it´s relative cheaper cost [11,13-15]. Both DTG and other older ARV has been associated with long term cardiometabolic toxicities [14,16-18]. However, not very much have been reported on the effectiveness and safety of the relatively DTG in Cameroon. However, some recent evidences show rapid weight gain associated with DTG based regiments [19-23]. It is therefore important to ensure a closer monitoring of viral suppression rates and safety of ARVs, with particular attention to DTG in ART programmes in Cameroon. In addition, for any new ART, there should always be a door for local real world studies to clarify whether results observed under clinical trials in other populations are also observed in everyday clinical practice in Cameroon PLHV.

### Research priority 2

Assess short- and long-term outcomes of ART in children and adolescents including physical and psychosocial development, morbidity and mortality, care outcomes from infancy to adolescence.

**Context:** despite the progress made towards the prevention of mother to child transmission (PMTCT) of HIV over decades, the mother to child (MTC) transmission of HIV remains relatively high in Cameroon, and the number of children diagnosed with HIV continue to rise, coupled with a rising mortality rate [24]. UNAIDS reported Cameroon amongst the 10 countries that make up 75% of all new paediatric HIV infections in 2015 [25] and WHO reported an alarming MTC HIV transmission rate of 12.8% in 2016 [26]. The trends seem not to have changed much over time. In 2019, 31 000 [24 000 - 38 000] Cameroonian children were living with HIV [27], and increase by just 1000 from 2011, suggestive of a very high mortality rate. A study in 2018 reported HIV as one of the leading causes of under-five mortality in Cameroon [28], and HIV has been associated with malnutrition in children, a leading cause of mortality in this group [29]. According to the Cameroon population based HIV impact assessment (CAMPHIA) report, children and adolescents are lagging behind adults in terms of ART coverage and viral suppression [30]. Reports on the long-term morbidity and mortality outcome of children on ART is relatively scarce. HIV has been associated with underweight and stunted growth as well as reduced cognitive development in children [31-34]. Understanding these factors could inform the provision of more holistic care to children living with HIV in Cameroon.

### Research priority 3

Assess the interactions and impact of HIV and ART on aging, major chronic diseases such as hypertension, cancer and diabetes mellitus, and age-related morbidities like osteoarthritis or osteoporosis.

**Context:** the scale up of antiretroviral therapy (ART) has dramatically increased the life expectancy of PLHV in Cameroon and across the globe. The longer life expectancy of PLHV is expected to change the demographics of the HIV epidemic, tilting the efforts towards dealing with the interactions between HIV, ART and major AIDS defining and non-AIDS defining comorbidities. The leading cause of death from HIV has moved from opportunistic infections to chronic diseases associated with aging [35-37]. Recent estimates in the US suggest that more than 70% of those with HIV will be over the age of 50 by 2030 [38]. Greater risk of cardiometabolic comorbidities, including diabetes and hypertension have been reported amongst older PLHV on ART [39-42], all of which are highly prevalent in Cameroon [27,43]. The growing population of people aging with HIV in Cameroon requires particular care in order to ensure early diagnosis and treatment, ensuring preventative measures for both AIDS and non-AIDS defining co-morbidities. Prioritizing research on intervention to improve quality of life and care, preventing and controlling chronic comorbid conditions is increasingly important as Cameroon HIV positive patients grow older.

### Research priority 4

Effectiveness of ART dispensation models and impact on short- and long-term ART adherence and retention: ART dispensation models including home based, Community based organization, private pharmacy, and multiple-month dispensation.

**Context:** the WHO recommended decentralisation and task-shifting as a core public health approach to enhance universal access to ART in resource-limited settings, where infrastructural and human resources are very limited [44]. More recent studies have reported compelling evidences that HIV treatment outcomes in nurse-led settings were comparable with those of physician-led ones, and effectiveness has also been demonstrated with community health workers [45-47]. The same light, decentralization of HIV care to primary care settings have been associated with comparable ART outcomes [48,49]. In same light, community dispensation of ART through community-based organizations (CBOs), community adherence clubs and HIV support groups were considered a promising and sustainable approaches to accelerating ART access in Cameroon. These models were adopted in the country´s strategic plan to accelerate and reinforce the provision of ART to all PLHV [50]. Cameroon piloted the community ART dispensation by CBOs in late 2016 and the model is gradually being scaled up in all the 10 regions of the country [51]. There is need to assess the short- and long-term impact of these community ART dispensation models on key ART outcomes such as ART adherence, ART retention and viral suppression.

### Research priority 5

National surveys and evaluations of HIV treatment and prevention programs including: HIV test and treat strategy, self-testing, Pre-exposure Prohylaxis, cost effectiveness of interventions, sentinel surveillance of HIV disease burden, national AIDS spendings, and integrated surveys on priority populations.

**Context:** national surveys are the best ways to have population-based estimates of important health parameters and constitute main drivers to informing evidence-based policies at national levels. National level HIV surveys and evaluations are necessary to establish baselines and evaluation of progress towards attaining strategic goals of the National AIDS Control Programme (NACC) as well as global goals. A few national health surveys have been fairly consistent in Cameroon, including the National Demographic and Health Surveys (DHS) which is done on a 7 years intervals, targets aspect on population-based prevalence of HIV, HIV testing, higher-risk sex as well as knowledge level of HIV prevention, prevention of mother-to-child transmission (PMTCT) [52]. The Cameroon population based HIV impact assessment was one of the major national HIV surveys which assessed progress towards the achievement of the HIV 95-95-95 targets, and highlighted HIV testing coverage and paediatric HIV care cascade as main areas requiring more efforts [30]. A national estimate on HIV viral suppression and drug resistant in 2015 reported alarming levels of virological failure and acquired HIV drug resistance in Cameroon, and recommended urgent need for better ART management, focused on improving ART adherence, availability of viral load monitoring, and more timely switches to second-line ART [9]. To informed national HIV strategies as well as their sustainability, more targeted nationwide surveys and evaluations of the effectiveness and efficiency and impact of existing national HIV strategies are needed. These include HIV self-testing, Pre-exposure prohylaxis, cost effectiveness of interventions, sentinel surveillance of HIV disease burden, national AIDS spendings, integrated surveys on priority populations.

Funding: the Kribi meeting was funded by the Cameroon research group of the Elizabeth Glaser Pediatric AIDS Foundation (EGPAF) and the International epidemiology Databases to Evaluate AIDS-Clinical Research Education Networking and Consultancy research group (CRENC-IeDEA).

## Discussion

The research priorities identified in this study represented a consensus from a majority of the participants, including HIV researchers, policy makers, implementing partners, clinicians and members of independent ethical review boards. Because capacity to support HIV research is finite, and with the need of investments in other competitive health research areas by the same few funding agencies, we must ensure that we build our sustainable capacity to initiate and pursue research of particular importance to our population. This initiative was unique in its nature and constitute the first effort to define HIV/AIDS research priority in Cameroon. The identified priorities generally align with the strategic priorities of the Cameroon National AIDS Control Programme, Kenyan National HIV research priorities, research priorities to inform treat all implementation in sub-Saharan Africa as well as WHO priority for adolescent HIV research [53-55]. The top research priority, which is focused on the effectiveness, safety and toxicity of new and old ART, is a global HIV research priority, especially in an era where most researchers are concerned about the short- and long-term toxicity of new drugs including DTG. It was in this light that the WHO HIV Department and the Special Programme for Research and Training in Tropical Diseases put in place a global database for active toxicity monitoring of antiretroviral (ARV) drugs to generate reliable evidence on the safety profile of new ARVs drugs including dolutegravir to address gaps in safety data [56]. Assessment of the effectiveness and close monitoring of ART drug resistance trends in Cameroon is critical, considering the reported alarming rates of drug resistance to the prior DTG ART regimens [9,10].

Current reports from UNAIDS and other international HIV research partners and stakeholders shows that paediatric and adolescent HIV care outcomes continue to lag behind adult HIV care outcomes [38,57]. This is coherent with the second research priority of this study, which focused on the short and long term paediatric and adolescent´s HIV outcomes. There´s an urgent need to focus limited research resources on this most needy children and adolescents living with HIV, with more emphasis on developing evidence based interventions to calve down the very high HIV morbidity, mortality and treatment failure rates in these children [10,28]. Research to understand and averting the negative impact of long term ART in children physical and cognitive development [31,32,34] are highly needed in Cameroon, if long term treatment of HIV in children is to be considered a positive phenomenon. In same light, rapidly increasing number of people aging with HIV and the shifting of the causes of HIV related deaths from opportunistic infections to age related comorbidities [35,36,58] is increasingly considered in the global HIV research agenda [59-61]. The research priority three which focussed on HIV and aging is therefore timely and consistent with global HIV research priorities. More research on the interaction between HIV, aging and age related comorbidities is particularly important in Cameroon, a country with a double burden of HIV and cardiometabolic comorbidities [43,62,63]. Within the context of the global HIV treat all strategy and attaining the 95-95-95 targets, the UNAIDS recommended broadening options for service delivery to reduce the burden on strained health systems and extend the reach of services, including greater use of community-based and rights-based approaches and new partnerships [64]. This is consistent with the research priority four, which focused on the effectiveness of ART dispensation models and impact on short- and long-term ART adherence and retention. Understanding how to maintain/strengthen the effectiveness hospital-based and community-based ARV dispensation, especially in a context of COVID-19 pandemic is crucial to maintain gains of years of the fight against HIV/AIDS as well as better outcomes for patients. In addition, identifying appropriate models of community ART dispensation with better ART outcomes in Cameroon is crucial to mitigate low usage of high-volume hospital in COVID-19 context and to inform and oriented the current scaling up community ART dispensation by CBOs as well as HIV support groups and adherence clubs. The fifth priority on national surveys and evaluations represents the overall needs of the Cameroon NACC to guide National HIV strategic planning and assess the effectiveness and cost effectiveness of existing national HIV strategies. This will help in reorientating and improving overall performance as well as ensuring sustainability of ongoing programs.

### Strengths and limitations

A major strenght in this formulation and refining of our five national major HIV research priorities is the multi-disciplinary nature of experts who were engaged and participated including implementing partners and stakeholders from 13 institutions, working on diverse aspects of HIV, ranging from HIV research, program implementation, HIV policy and management, care delivery, and ethical review boards. The use of the Delphi´s approach, an approach with proven effectiveness in achieving consensus on health research priorities is the second major strenght. Thirdly, it is worth mentioning the active participation of more 85% of the participants in more than one round in the process reflected an effective coordination of the process, which is often a challenge in using the Delphi´s approach [65]. Fourthly, we used two different approaches with 80% concordance to rank the research priorities, and this further affirms the internal validity of the process. Last but not the least, our approach benefited from a direct involvement and support of the ministry of health which is the major stakeholder coordinating all activities for reaching the 95-95-95 goals. Notwithstanding, it is always possible that the process could have introduced some biases, especially as the final consensus could been unconsciously influenced by those with stronger opinions, especially during the larger meeting that was characterised by interactive open debates. Secondly, other stakeholders including the civil society and representatives of people living with HIV were not engaged at the beginning of the process. Finally, these research priorities are based on current knowledge about the pandemic and the science to prevent, treat, and ultimately cure HIV, we cannot rule out the possibility that COVID-19 pandemic may have impacted on years gain before the pandemic and therefore creating a more important gap to be filled.

## Conclusion

The Cameroon HIV research priorities generated during the Kribi conference using the Delphi approach process resulted from the consensus of a broad group of individuals engaged in accelerating the ‘treat all´ HIV policy and organized in the CAM-HERO consortium. This initiative was directly under the coordination of the Cameroon Ministry of Health, through its Division of Health Operational Research (DROS) and the National AIDS control committee (NACC) and highlighted critical areas of inquiry with potential relevance for the nation and for funders, for the regulatory bodies, and other programme strategies. It is therefore our deep hope that these priorities will guide the acceleration needed to meet the 95-95-95 goals in the entire nation, despite the COVID-19 pandemic.

### What is known about this topic

- The Treat-All remains the globally endorsed approach to attain the 95-95-95 targets and end the HIV/AIDS pandemic by 2030; Cameroon implemented the HIV Treat-All strategy nationwide in 2016;
- In the 2018 Cameroon population-based HIV impact assessment (CAMPHIA) report, only 46.9% of people living with HIV/AIDS (PLHV) knew their status, 91.3% of those who knew their status were on ART and 80.0% of those on ART had viral suppression;
- There was no country-specific research agenda to inform strategies for improving Cameroon national HIV policy.

### What this study adds

- The Treat-All remains the globally endorsed approach to attain the 95-95-95 targets and end the HIV/AIDS pandemic by 2030; Cameroon implemented the HIV Treat-All strategy nationwide in 2016;
- In the 2018 Cameroon population-based HIV impact assessment (CAMPHIA) report, only 46.9% of people living with HIV/AIDS (PLHV) knew their status, 91.3% of those who knew their status were on ART and 80.0% of those on ART had viral suppression;
- There was no country-specific research agenda to inform strategies for improving Cameroon national HIV policy.
